# Acute Flaccid Paralysis by Enterovirus D68 Infection: First Italian Description in Adult Patient and Role of Electrophysiology

**DOI:** 10.3389/fneur.2017.00638

**Published:** 2017-11-27

**Authors:** Marco Ceccanti, Emilia Sbardella, Federica Letteri, Manuela De Michele, Anne Falcou, Federica Romanzi, Emanuela Onesti, Maurizio Inghilleri

**Affiliations:** ^1^Department of Neurology and Psychiatry, Sapienza University, Rome, Italy; ^2^Emergency Department, Stroke Unit, Policlinico Umberto I, Sapienza University, Rome, Italy

**Keywords:** acute flaccid paralysis, enterovirus D68, electrophysiology, nerve conduction study, EMG, poliomyelitis, bone marrow transplantation

## Abstract

A Peruvian woman was admitted to the Emergency Department, due to an acute flaccid paralysis (AFP) of the upper limbs that progressively involved also lower limbs and respiratory muscles. She previously suffered from non-Hodgkin’s lymphoma and had to undergo hematopoietic stem cell transplantation. A magnetic resonance imaging showed a T2 hyperintensity in the anterior and central region of the cervical segment with an elective involvement of gray matter. This finding, combined with other clinical, laboratory, and electrophysiological data, led to a diagnosis of AFP. Enterovirus D68 was isolated in the patient’s cerebrospinal fluid, plasma, and throat swab. To our knowledge, this is the first Italian case of AFP by Enterovirus D68 infection in an adult. The diagnostic assessment and management of AFP by Enterovirus D68 are discussed.

## Introduction

Poliomyelitis is an acute, febrile illness caused by a wild-type poliovirus infection. Disease is characterized by aseptic meningitis and weakness or paralysis of one or more extremities. Following the widespread use of polio vaccine, the incidence of poliomyelitis dramatically decrease in the western hemisphere and international eradication programs are making progress in the rest of the world. Nevertheless, sporadic cases of acute paralysis similar to poliomyelitis also occur with other enterovirus serotypes. In particular, Enterovirus D68 (D68V) has also been implicated in rare cases of acute paralysis in the United States (1–3). In the setting of a 2014 surge of respiratory illnesses due to D68V, there were reports of children with acute focal limb weakness and/or cranial nerve dysfunction, with a mild-to-moderate lymphocytic pleocytosis in the cerebrospinal fluid (CSF) and gray matter spinal cord lesions on magnetic resonance imaging (MRI), similar to poliomyelitis (4, 5). D68V was identified in nasopharyngeal specimens of a subset of these patients, but not from the CSF of any cases.

Acute flaccid paralysis (AFP) is the name used for a wide spectrum of neuromuscular diseases, ranging from acute inflammatory motor polyneuropathy to hypo/hyperkalemic paralysis and poliomyelitis and polio-like infections (6–9). Despite the widespread use of polio vaccine, sporadic cases of acute paralysis similar to paralytic poliomyelitis also occur with other enterovirus serotypes. In particular, Enteroviruses are small, single-stranded RNA viruses of the Picornaviridae family that share similar morphologies, structures, molecular properties, and replication strategies. They are commonly involved in both acute and chronic cardiac disease, hand, foot, and mouth disease, respiratory infections, herpangina, myositis, pleurodynia, eye infections, including acute haemorrhagic conjunctivitis, encephalitis, aseptic meningitis, and AFP (10). D68V was first described in California in 1962 in four children with severe respiratory tract infection and pneumonia; reports of D68V since then have been infrequent, with only 699 diagnoses being confirmed worldwide up to 2014, most of which induced pulmonary infections and sometimes AFP in children (11). Numerous cases, which caused severe respiratory illness in asthmatic children but were rarely associated with AFP, were described in 2014 in the USA. Since then, many D68V infections have been reported in Canada, Europe, and Asia, sometimes in association with AFP. Only one case of D68V associated with AFP has been described in Italy (11, 12). Some authors have described infections in Italy before (13) and after (14, 15) the 2014 outbreak, all of which were associated with severe respiratory symptoms. The comparative genomic analysis showed that most of the D68V strains circulating in the 2014 outbreak in the USA differed significantly from prior strains (16), thereby indicating a viral evolution (17).

Immunocompromised hosts, such as patients with hematological malignancies receiving chemotherapy, including hematopoietic stem cell transplant (HSCT) recipients, are susceptible to viral infection complications. To date, six cases of D68V infection have been reported in adult patients with hematological malignancies who had undergone HSCT (18); all of these patients had respiratory diseases, and one was paraplegic as a result of a compressive vertebral fracture. No cases of AFP have been reported.

We report the first case of AFP due to D68V in an adult transplanted patient affected by diffuse large B-cell non-Hodgkin’s lymphoma. Written informed consent was obtained from all the people whose identities could be revealed by data included in this case report.

### Clinical Case

In April 2011, a 50-year-old Peruvian female who had been living in Italy for many years came to the hematology center of Policlinico Umberto I in Rome due to a bilateral inguinal lymphadenopathy. The diagnostic protocol led to a diagnosis of follicular non-Hodgkin’s B lymphoma (B-NHL) in stage IV of the disease. She underwent six cycles of GA101 (obinutuzumab)-CHOP21 (cyclophosphamide, doxorubicin, vincristine, and prednisone), followed by five doses of GA101 (19), with complete remission. In November 2012, the patient presented a relapse of the disease, which had evolved into a diffuse large B-cell lymphoma. She received four cycles of the R-DHAP (rituximab, dexamethasone, cytarabine, cisplatin) treatment regimen (20), with a good response. In June 2013, she underwent an auto-HSCT after receiving the FEAM (fotemustine, etoposide, cytarabine, melphalan) conditioning regimen (21). Six months after the auto-HSCT, owing to progression of the disease in the right humerus, she received local radiotherapy (total dose 30 Gy) and four cycles of the IEV protocol (epirubicin, ifosfamide, and etoposide) (22), which yielded a partial response. Since the patient had an HLA-matched family donor, in December 2014, she underwent an allo-HSCT after receiving a reduced intensity conditioning regimen with rituximab, cyclophosphamide, fludarabine, and thiotepa, according to the BCNHL protocol (23). Cyclosporine and methotrexate were used as the graft versus host disease (GVHD) prophylaxis.

After the transplant, the patient presented acute GVHD, with skin, liver, and gut involvement (grade IV), treated with high-dose corticosteroids and extracorporeal photopheresis. The GVHD evolved into a severe chronic GVHD. During this period, the patient displayed persistent CMV DNA-emia despite antiviral treatment with ganciclovir and foscarnet. The monitoring of CD4+ lymphocytes in the peripheral blood showed a count persistently below 200/μl. In July 2016, she developed a Gram-negative sepsis, which resolved in 1 month. A low-grade fever persisted.

In early October 2016, the patient was admitted to the hematological Emergency Department (ED) of Policlinico Umberto I in Rome after developing an acute proximal weakness of the left arm, with an impairment in lifting the same limb. She underwent a Doppler ultrasound of the left upper limb, which did not show any vascular involvement. Brain Computed Tomography and MRI were normal. As the strength deficit was mild [4/5 on the Medical Research Council (MRC) scale], she was discharged. The day after, the patient worsened, displaying a total impairment in arm elevation; a similar deficit had also extended to the proximal segment of the contralateral arm. A dropped head syndrome also occurred within a few hours. She was admitted to the central ED of our hospital.

The first neurological consultation revealed ptosis of the right eye lid, a severe motor deficit in the proximal muscles of the upper limbs (2/5 MRC scale, bilaterally), and a mild motor deficit in the distal muscles (4/5 MRC scale, bilaterally). Lower limb function was normal. There was no evidence of a sensory deficit. Deep tendon reflexes were absent in the upper limbs and normal in the lower limbs. Hoffmann sign was absent. The flexor plantar reflex was present bilaterally. Voluntary cough was feeble. Clinical features were suggestive of a man-in-the-barrel syndrome (24).

Considering the acute development of a motor deficit with no deep tendon reflexes in the upper limbs and the absence of sphincter involvement, a lumbar puncture was performed to exclude an acute inflammatory polyneuropathy, as were electrophysiological assessments to exclude neuromuscular junction diseases such as myasthenia gravis or botulism. In the meantime, 8 ml of CSF were collected for the physicochemical examination and microbiological assessments. A brain and spinal cord MRI was performed. The blood tests were normal except for a high C-reactive protein (12.99 mg/dl).

## Diagnostic Assessments

The first lumbar puncture revealed mild pleocytosis with 17 cells/μl (9 monocytes, 8 neutrophils). Other parameters were normal. CSF was tested for common neurotropic viruses by routine polymerase chain reaction (PCR) assays.

**Figure 1:**
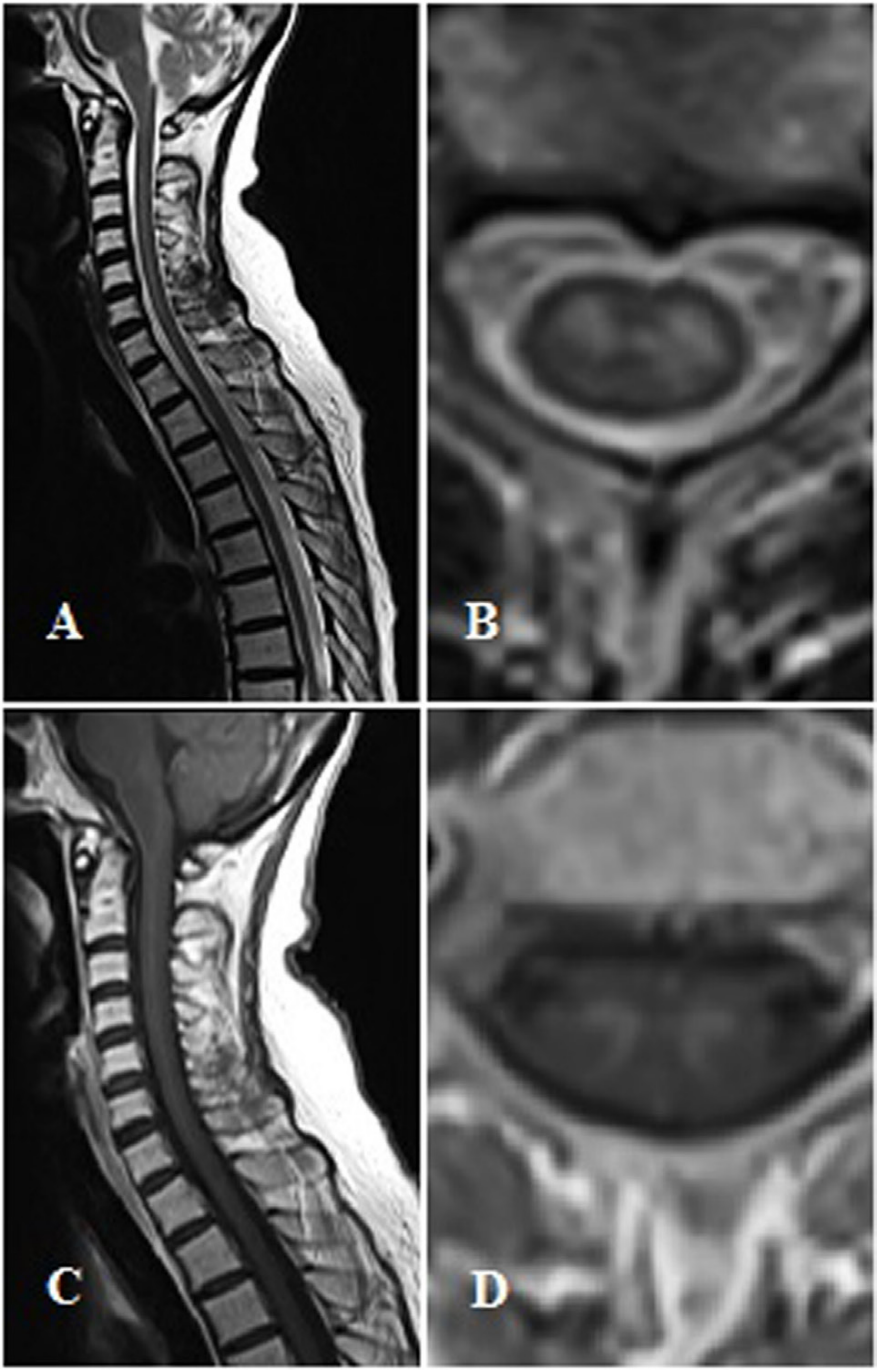
Magnetic resonance imaging findings. Panel **(A,B)** show T2-weighted sagittal and axial images. Panel **(C,D)** show T1-contrast sagittal and axial images.

Spinal cord MRI revealed a swelling in the C2-T1 tract, with a T2 hyperintensity in the central region that enhanced after gadolinium injection. Imaging data were consistent with ischemic damage or medullary localization of a lymphoma. A second opinion of the MRI scans revealed an elective T2-hyperintensity and gadolinium enhancement of the medullary gray matter, mainly involving the anterior horns (Figure 1). Steroid therapy with dexamethasone, ceftriaxone, and intravenous acyclovir was started.

An electrophysiological assessment was performed 4 days after admission. Owing to the weakness of the cough, the phrenic nerves were explored. Cathodal stimulation was supra-maximally performed with a monopolar needle electrode positioned behind the sternocleidomastoid muscle, at the level of the cricoid cartilage. The anode was placed on the sternal manubrium (25). Compound motor action potential (cMAP) was recorded bilaterally using Ag/Cl electrodes placed over the eighth intercostal space, at the costochondral junction, referred to the opposite side. Electrophysiology yielded normal values for sensory nerve conduction as well as for the cMAP amplitude from the ulnar and median nerves bilaterally and the absence of *F*-waves. Motor conduction and *F*-waves in the lower limbs were normal. cMAP amplitudes from the phrenic nerves were below 30% of lower normal values. Data are shown in Table 1.

**Table 1:** Electrophysiological assessments.

	R	L	Control values
**Sensory NCS**			
SCV (m/s)			
Sural	48.0	45.0	>39.5
Ulnar	45.0	48.0	>45.0
Amplitude (μV)			
Sural	16.3	15.8	>6.3
Ulnar	11.2	10.5	>7.5
**Motor NCS**			
DML (ms)			
Medial plantar	4.3	4.1	<4.8
Ulnar	2.5	2.2	<2.8
Median	2.8	2.9	<3.2
Phrenic	9.3	9.8	<9.5
MCV (m/s)			
Medial plantar	46.0	48.0	>40.3
Ulnar	55.0	53.0	>50.4
Median	52.4	55.3	>51.4
Distal/proximal cMAP amplitude (mV)
Medial plantar (A/K)	11.2/11.0	12.3/11.9	>8.7
Ulnar (W/UE/AE/EP)	9.8/9.0/–/–	8.7/7.8/–/–	>8.3
Median (W/E/EP)	13.1/12.7/–/–	12.6/11.8/–/–	>9.0
Phrenic	0.2	0.3	>0.9
*F*-waves (ms)			
Medial plantar	48.7	47.9	<54.3
Ulnar	n.d.	n.d.	<27.6
Median	n.d.	n.d.	<26.8
**SEP**			
Latency (ms)			
Median: N20	16.2	17.0	<20.3
P25	18.9	19.5	<23.7
Tibial P40	40.8	39.9	<42.1
N45	45.0	43.3	<47.6
Amplitude (μV)			
Median: N20-P25	2.2	2.4	>1.5
Tibial P40-N45	0.8	1.0	>0.6

*NCS, nerve conduction study; SCV, sensory conduction velocity; DML, distal motor latency; MCV, motor conduction velocity; SEP, sensory-evoked potential; R, right; L, left; n.d., not detectable; A, ankle; K, knee; W, wrist; UE, under elbow; AE, above elbow; EP, Erb point; cMAP, compound motor action potential*.

Repetitive nerve stimulation did not reveal any pre- or postsynaptic alteration in the neuromuscular junction safety factor, thus excluding myasthenia gravis and botulism. Electromyography did not detect any fibrillary elements; some satellite potential was present in the upper limb muscles, together with severe spatial recruitment deficit, with a proximal–distal gradient. Characteristically, doublets and triplets were observed in the upper limbs muscles. Needle electromyography from the diaphragm muscle was not performed.

Somatosensory-evoked potentials (SEP) were performed on the tibial and median nerves to confirm the lack of sensory signs that emerged at the clinical examination. Latency, amplitude, and morphology were normal bilaterally, thus confirming no involvement of the posterior columns.

In view of the results yielded by the electrophysiology, which suggested acute anterior horn damage, a second lumbar puncture was performed. CSF and plasma samples for virus PCR [cytomegalovirus (CMV), Epstein–Barr (EBV), enterovirus A-D, herpes simplex (HSV) 1 and 2, human immunodeficiency virus (HIV), human herpesvirus 6-(HHV 6-), parechovirus, varicella zoster virus (VZV), and flavivirus] and IgM/IgG antibodies (anti-polio 1, 2, and 3, West Nile, Coxsackie A and B 1–6, echovirus, adenovirus) and cytological data were collected, as were rectal and throat swabs.

The CSF analysis revealed a marked pleocytosis with 130 cells/μl (125 monocytes and 5 polymorphonucleocytes), but normal glucose and protein levels. The cytological analysis did not detect any malignant lymphocyte in CSF. The viral analysis identified a D68V in the CSF, plasma, and throat swab. All other viral markers were negative, except for the mild presence of CMV-DNA in plasma.

Despite the reduction in cMAP amplitude in the phrenic nerves and the weakness of cough, the normal arterial blood gas values ruled out the need for an elective tracheotomy. However, 6 days after admission to hospital, the patient suffered from an acute respiratory failure and immediately underwent a tracheotomy. A chest X-ray showed the elevation of the hemi-diaphragms, thus confirming phrenic nerve involvement.

Intravenous immunoglobulins (IVIg) were dispensed as replacement therapy and passive immunization, as previously suggested (26–28), though without any clinical effect. The patient then developed a severe pancytopenia with a non-specific hypocellular marrow, as demonstrated by a biopsy. The complete blood count data are shown in Table 2. The patient underwent numerous blood component transfusions and received a bone marrow stimulant (filgrastim). Unfortunately, she passed away 17 days after being admitted to hospital.

**Table 2:** Complete blood count.

	Day 1	Day 3	Day 4	Day 4/2	Day 9
RBC	4,580	3,780	3,020	2,730	–
WBC	5,250	5,020	2,070	1,550	–
PLT	288	192	112	95	5

*Days after admission to hospital*.

*RBC, red blood count; WBC, white blood count; PLT, platelet*.

## Discussion

This is the first report of AFP induced by D68V in an adult stem cell transplanted patient in Italy. Only one child with AFP associated with D68V has previously been described in this country (12), though the authors of that report were unable to isolate the virus in the CSF. Most of the infected patients with D68V described previously were asthmatic children (11).

A number of differential diagnoses were considered. The patient’s clinical history was consistent with non-Hodgkin’s B lymphoma; the medullary localization of the disease was considered at first, though the specific involvement of medullary gray matter was unlikely because invasion of the spinal extradural space is the most frequent feature. Moreover, the thoracic segment is usually predominantly affected (29), and the cytological analysis did not reveal any malignant lymphocytes in the CSF.

A diagnosis of acute motor axonal neuropathy (AMAN) was also considered. MRI may highlight root enhancement in AMAN (30), whereas T2-hyperintensity and gadolinium enhancement of the spinal cord gray matter have not been reported. Nevertheless, other authors have reported myelitis associated with AMAN (31, 32), though clinical sensory impairment, sphincter dysfunctions, and Babinski sign, as well as a SEP impairment, are expected to accompany neuropathic signs. The marked pleocytosis, especially in the second lumbar puncture, was suggestive of an infectious disease.

All the clinical and diagnostic findings pointed to a prevalent anterior horn involvement, even though the MRI revealed some T2-hyperintensity and gadolinium enhancement even in the posterior horns. The posterior, lateral, and ventral columns were clinically preserved, thus suggesting a marked tropism of the virus for gray matter.

The electrophysiological assessment confirmed this finding. SEP normality attested to the integrity of the peripheral and central somatosensory tracts. Conduction studies, performed some days after the clinical onset of the symptoms, yielded normal cMAP amplitude values in the upper limb nerves and the absence of *F*-waves, as is observed at the onset of Wallerian degeneration. These data were confirmed by needle EMG, which detected a reduced spatial recruitment. Fibrillation and positive sharp waves were not found, probably owing to the early timing of the examination. Doublets and triplets, often associated with both degenerative and inflammatory motor neuron diseases (25), were found.

The clinical assessment and laboratory, electrophysiological and MRI findings were in keeping with the diagnosis of poliomyelitis, initially made on the basis of the inflammatory involvement of gray matter in the spinal cord. Neurotropic pathogens associated with AFP, such as enterovirus A-D, EBV, HSV1 and 2, HIV, HHV 6, parechovirus, varicella zoster, flavivirus, poliovirus, West Nile, coxsackie A and B 1–6, echovirus and adenovirus, were investigated. PCR for CMV did detect this virus in plasma though not in the CSF. Enterovirus D, later characterized as D68V, was isolated in plasma, the throat swab and CSF. The presence of this neurotropic virus in biological fluids, together with the characteristic polio-like clinical and diagnostic findings, suggest that D68V was the cause of the patient’s disease.

The phrenic nerve assessment proved to be a reliable means of predicting respiratory failure, as previously demonstrated in ALS patients (33–35), particularly when spirometry is not possible owing to limitations due to the illness (36). The chest X-ray and subsequent restrictive failure confirmed the diaphragmatic weakness. The arterial blood gas examination did not reveal any increase in pCO2 or reduction in pO2. We may speculate that other accessory respiratory muscles, such as intercostals, sternocleidomastoid, and trapezius muscle, which are innervated by nerves not originating in the C2-T1 tract, temporarily compensated for the diaphragmatic deficit. Phrenic nerve assessment, when supported by clinical and imaging data, may be used to help to decide how urgently needed a respiratory support is in neuromuscular diseases. Technical expedients, such as using needle stimulation, are required to avoid submaximal stimulation, which could give rise to misdiagnoses.

Other authors have described infections caused by D68V in patients with hematological malignancies or in HSCT-recipients presenting respiratory symptoms (18). D68V apparently shares tropism with poliovirus. Recent evidence (37) suggests that human pluripotent hematopoietic stem cells express CD155, a poliovirus receptor, and that poliovirus infects and replicates in human hematopoietic progenitor cell lines. This might explain the pancytopenia found in our patient. Moreover, we may speculate that the latent virus was transmitted through HSCT and started replicating after optimization of the immunosuppressive therapy due to the GVHD. Unfortunately, a viral assessment was not performed on the bone marrow biopsy.

Our patient did not respond to IVIg treatment. IVIg is commonly used in passive immunization or replacement immunotherapies in enterovirus infections in neonates and immunocompromised patients (26–28). The effectiveness of IVIg treatments for enterovirus infections depends on the presence and level of anti-enterovirus neutralizing antibodies that are specific for the infecting serotype (27, 38, 39). IVIg treatment was also used for type 3 vaccine-derived poliovirus infection in an immunodeficient Iranian child (40), though to little avail. By contrast, IVIg has proved effective in West Nile virus infections (41–45).

A recent study (46) showed that the 2014-D68V strain had some unique antigenic sites other than the prototype site. The authors of that study detected high titers of neutralizing antibodies against both the prototype and 2014 strains, but significantly lower titers against the most recent strain in all IVIg lots they tested. However, it should be borne in mind that that study was performed in the US, where the 2014 outbreak started, and that US donors are more likely to be immunized against the 2014 strain and, consequently, to have a more effective IVIg than Italian donors. An evaluation of enterovirus neutralizing antibodies from IVIg lots collected in different countries is needed.

## Conclusion

This is the first description of AFP induced by D68V in an adult patient in Italy. D68V infection represents an emerging acute illness whose clinical course may resemble that of the polio infection. Physicians working in the emergency setting need to be aware of this potentially life-threatening disease. A multidisciplinary approach is required for the diagnosis, which may be challenging. In particular, in our patient, the electrophysiological assessment highlighted the need to search for a polio-like virus. Interestingly, a reduction in cMAP amplitude in the phrenic nerves, when associated with clinical evidence of weakness of cough, may provide the indication for respiratory support.

## Ethics Statement

This article does not contain the results of any studies performed by the authors on humans or animals. Additional informed consent was obtained from all the people whose identity may be disclosed by information included in this article.

## Author Contributions

MC, FR, EO, and MI: writing of the paper and electrophysiological studies. ES, MM, AF, and FL: management of the patient during hospitalization in the ED.

## Conflict of Interest Statement

The authors declare that the research was conducted in the absence of any commercial or financial relationships that could be construed as a potential conflict of interest.
